# Case Report: Expanded dengue syndrome with acute pancreatitis and pericardial effusion in octogenarians, from recovery to fatal shock

**DOI:** 10.3389/fmed.2026.1761522

**Published:** 2026-03-02

**Authors:** Chen-Hsuan Lin, Li-Teh Liu, How-Chen Wang, Chia-Hsin Chang, Jih-Jin Tsai

**Affiliations:** 1 School of Medicine, College of Medicine, Kaohsiung Medical University, Kaohsiung City, Taiwan; 2 Department of Medical Laboratory Science and Biotechnology, College of Medical Technology, Chung Hwa University of Medical Technology, Tainan City, Taiwan; 3 Department of Medical Education, Kaohsiung Medical University Hospital, Kaohsiung City, Taiwan; 4 Tropical Medicine Center, Kaohsiung Medical University Hospital, Kaohsiung City, Taiwan; 5 Division of Infectious Diseases, Department of Internal Medicine, Kaohsiung Medical University Hospital, Kaohsiung City, Taiwan

**Keywords:** acute pancreatitis, case report, dengue fever, DENV-2, expanded dengue syndrome, pericardial effusion

## Abstract

Severe dengue fever (DF) can involve multiple organs, yet acute pancreatitis (AP) and pericardial effusion remain rare and easily overlooked, particularly in elderly patients who often present atypically. This report describes two women in their eighties who developed both complications, underscoring the diagnostic challenges and clinical importance of recognizing these manifestations early. Clinical data, laboratory results, and imaging findings were reviewed retrospectively. The first patient initially presented with fever and urinary symptoms before progressing to severe DF with shock, thrombocytopenia, acute kidney injury, and altered consciousness; computed tomography later confirmed AP and pericardial effusion, and she recovered with supportive care. The second patient presented with out-of-hospital cardiac arrest and multiorgan failure, with imaging demonstrating AP and pericardial effusion consistent with dengue shock syndrome; despite aggressive resuscitation, she died on hospital day two. These cases highlight that dengue-associated AP and cardiac serositis may mimic other acute illnesses or coexist with comorbid conditions in older adults, delaying diagnosis. Early imaging, point-of-care ultrasonography, and close hemodynamic monitoring are essential to identify organ involvement promptly and improve outcomes in this vulnerable population.

## Introduction

Dengue fever (DF) is a mosquito-borne viral infection caused by the dengue virus (DENV) and transmitted primarily by Aedes mosquitoes. Under the 2009 World Health Organization (WHO) classification, DF is categorized as dengue with or without warning signs and severe dengue, the latter defined by plasma leakage, major bleeding, or severe organ involvement such as hepatitis, central nervous system dysfunction, cardiac impairment, or acute kidney injury (1). While most infections are self-limited, DF can present atypically, particularly in older adults or those with comorbidities.

In 2011, the WHO introduced the concept of Expanded Dengue Syndrome (EDS) to encompass multi-organ involvement beyond classical dengue manifestations, including neurologic, gastrointestinal, renal, respiratory, and cardiovascular complications (2). These atypical presentations often pose diagnostic challenges and may mimic other acute illnesses.

Acute pancreatitis (AP) is an uncommon but recognized complication of DF. Although gallstones, alcohol, and hypertriglyceridemia account for most AP cases in the general population, infectious etiologies contribute up to 10% (3). Dengue-associated AP is rare, with reported incidence ranging from 0.8 to 7% (4), and mechanisms proposed include direct viral injury, immune-mediated inflammation, and ischemia related to severe plasma leakage or shock (5).

Cardiac involvement is another notable complication of DF. Reported manifestations range from asymptomatic electrocardiographic changes to myocarditis and pericardial effusion. Pericardial effusion, in particular, is increasingly recognized as a marker of severe dengue and is thought to reflect cytokine-mediated endothelial dysfunction and increased vascular permeability (6). Although usually self-limiting, its presence warrants close monitoring.

We report two elderly patients with DF complicated by both AP and pericardial effusion, one of whom died despite intensive management. These cases underscore the importance of recognizing atypical and severe dengue manifestations—especially in high-risk populations—and highlight the need for early diagnosis and tailored supportive care to improve outcomes.

## Case presentation

### Patient 1

An 81-year-old woman with diabetes mellitus, hypertension, hyperlipidemia, and prior ischemic stroke initially presented with high fever and weakness. Laboratory tests showed elevated lipase (144 U/L), hyperglycemia, and pyuria; *Escherichia coli* grew in urine culture. She declined admission and was treated for presumed urinary tract infection.

On post-symptom onset (PSO) day 4, she returned with worsening weakness, abdominal and chest discomfort, and tachypnea. Repeat evaluation revealed leukocytosis, severe thrombocytopenia (9,000/μL), acute kidney injury (creatinine 4.22 mg/dL), diabetic ketoacidosis, and lactic acidosis—findings disproportionate to an isolated urinary tract infection. Given the rapid clinical deterioration and thrombocytopenia in an endemic setting, dengue infection was suspected. Dengue nonstructural protein 1 (NS1) antigen test was positive, and polymerase chain reaction (PCR) confirmed DENV-2 infection. Serology also demonstrated positive IgG antibodies, consistent with secondary dengue infection. Chest radiography showed a right pleural effusion. She was admitted to the intensive care unit for severe dengue (as defined by shock, thrombocytopenia, and severe organ involvement) and received fluids, platelet transfusion, metabolic correction, and empiric ceftriaxone.

**Figure 1:**
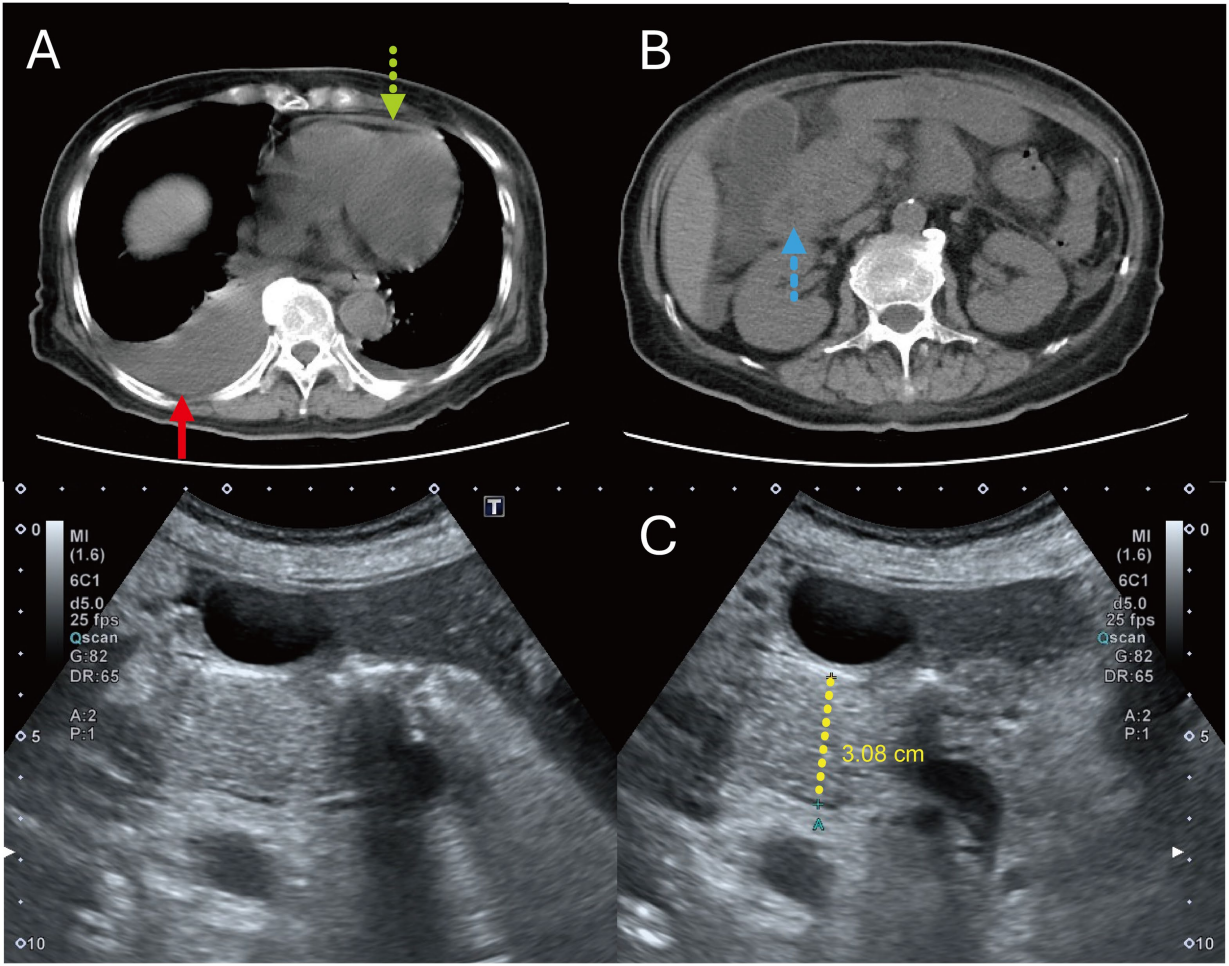
Patient 1, non-contrast CT (PSO 7) and transabdominal ultrasonography (PSO 13). **(A)** Non-contrast abdominal CT on PSO 7 revealed a pericardial effusion (green dotted arrow) and left and minimal right pleural effusions (red arrow). **(B)** A 3.3-cm iso-dense lesion at the pancreatic head with surrounding inflammatory edema (blue dashed arrow). **(C)** Follow-up transabdominal ultrasonography on PSO 13 demonstrated a 3.08-cm isoechoic lesion at the pancreatic head (yellow dotted line).

By PSO 6, she developed altered consciousness, diffuse ecchymoses, edema, and coffee-ground vomiting. Non-contrast abdominal computed tomography (CT) revealed pancreatic head enlargement with surrounding inflammation, ascites, right pleural effusion, and pericardial effusion, consistent with AP and plasma leakage (Figures 1A,B). Antibiotics were adjusted based on culture results. Anemia required packed red blood cell transfusion and proton pump inhibitor therapy.

Given pancreatic swelling and mildly elevated tumor markers, malignancy was initially suspected. Ultrasonography (PSO 13) (Figure 1C) demonstrated a 3.08-cm isoechoic lesion at the pancreatic head, but subsequent endoscopic ultrasonography (PSO 35) showed no mass lesions. Her condition gradually stabilized, and she was discharged on PSO 38 with a final diagnosis of severe dengue complicated by AP, pleural effusion, and pericardial effusion. Lipase levels normalized 2 months later.

### Patient 2

An 83-year-old woman with diabetes mellitus, hypertension, and coronary artery disease was brought to the emergency department after an out-of-hospital cardiac arrest. According to her family, she had received a COVID-19 vaccine 3 days prior and subsequently experienced fever and generalized malaise. Return of spontaneous circulation was achieved, but she remained comatose.

Initial laboratory evaluation showed severe thrombocytopenia (24,000/μL), metabolic acidosis, hyperlactatemia (17.39 mmol/L), hyperglycemia, elevated liver enzymes, hyperammonemia, and impaired renal function. Amylase and lipase were elevated (120.6 and 274.8 U/L). Dengue NS1 and PCR were positive for DENV-2. Serologic testing revealed dengue IgG positivity, indicating secondary infection. Chest radiography showed right pleural effusion; electrocardiography revealed low limb-lead voltage.

**Figure 2:**
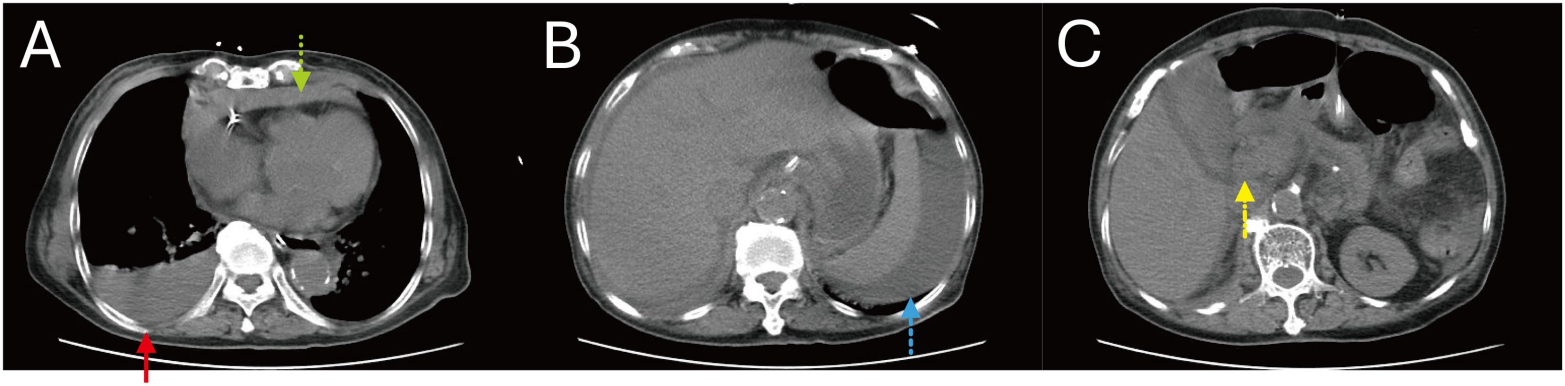
Patient 2, non-contrast abdominal CT at presentation. **(A)** Non-contrast abdominal CT at presentation showed a pericardial effusion (green dotted arrow), right pleural effusion (red arrow), **(B)** perisplenic ascites (blue dashed arrow), and **(C)** pancreatic head enlargement with peripancreatic inflammatory infiltration (yellow dash-dotted arrow).

Abdominal CT demonstrated pancreatic swelling with peripancreatic infiltration, gallbladder wall edema, ascites, right pleural effusion, and a pericardial effusion, suggestive of AP and severe plasma leakage (Figure 2). She was diagnosed with severe dengue manifesting as dengue shock syndrome (DSS) and AP.

Despite aggressive resuscitation with intravenous fluids, vasopressors, blood products, and broad-spectrum antibiotics, she developed refractory shock and hypoxemia and died on hospital day 2.

## Discussion

These two cases illustrate the diagnostic and clinical challenges of severe dengue complicated by AP and pericardial effusion—an uncommon but clinically important constellation of findings. Although AP has been reported in up to 7.7% of DF cases (7), most published patients are younger adults (8), typically presenting with epigastric pain during the febrile or critical phase (9). In contrast, both of our patients were women in their eighties who presented with nonspecific features, highlighting that elderly individuals may manifest severe dengue or EDS atypically, often without classical abdominal pain or warning signs. This observation is consistent with studies showing that older adults have blunted febrile responses, fewer mucocutaneous findings, and a higher risk of severe disease and organ dysfunction.

In this context, early dengue infection may mimic or coexist with other infectious processes, particularly in elderly patients who often present atypically. In Case 1, an initial diagnosis of urinary tract infection was reasonable; however, the rapid progression to severe thrombocytopenia, metabolic acidosis, shock, and radiographic evidence of plasma leakage was disproportionate to an isolated bacterial infection and prompted reconsideration of the diagnosis. Clinicians should suspect dengue in patients with acute febrile illness who develop unexplained thrombocytopenia or serosal effusions, even when an alternative diagnosis has been established. Although dengue is a viral illness, empiric antibiotic therapy may be appropriate in severe dengue when bacterial coinfection or sepsis cannot be confidently excluded, particularly in elderly patients with shock or positive cultures (10). Antibiotic use should be reassessed and de-escalated once bacterial infection is excluded.

The pathophysiology of dengue-associated AP is likely multifactorial, involving direct viral invasion of pancreatic tissue, pancreatic ductal obstruction from inflammatory edema, and immune-mediated injury (3). These mechanisms may converge in older adults with diabetes or hypertension, whose pancreatic microvasculature and immune response are already compromised, thereby increasing susceptibility to organ-specific injury. Furthermore, severe dengue is often driven by antibody-dependent enhancement (ADE), where pre-existing heterologous antibodies from a primary infection facilitate viral entry into host cells during a secondary infection (11). This process leads to an amplified immune response, increased viral load, and massive cytokine release, which contribute to severe plasma leakage and multiorgan dysfunction. Given that both patients were infected with DENV-2, a serotype frequently implicated in severe disease, ADE may have played a significant role in their rapid clinical deterioration. Importantly, AP may mimic other critical-phase complications of DF—such as hepatitis, shock, or gallbladder edema—making timely diagnosis difficult when abdominal pain is absent or overshadowed by systemic instability.

Management of dengue-related AP remains largely supportive. Fluid therapy must balance the need for pancreatic perfusion with the risk of worsening plasma leakage in severe DF. Invasive procedures are rarely indicated unless complications such as necrosis, pseudocysts, or abscess occur (12). Patient 1’s prolonged enzyme elevation raised concern for such sequelae (13), although follow-up imaging excluded these complications. Her favorable recovery after conservative management reinforces that early recognition and careful hemodynamic titration are crucial in preventing progression. Managing fluid resuscitation in octogenarians with severe dengue is particularly challenging. While aggressive crystalloid administration is standard for treating DSS (14), elderly patients with pre-existing diabetes, hypertension, and coronary artery disease have reduced cardiac compliance and impaired renal compensation. In these cases, excessive fluid may exacerbate third-space losses, such as the pleural and pericardial effusions observed in our patients, potentially leading to acute heart failure or respiratory distress (14). Therefore, fluid management in this population should emphasize a judicious, titratable strategy guided by frequent reassessment rather than standard weight-based boluses.

Cardiac involvement represents another key dimension of severe dengue. Pericardial effusion is reported in up to 7.4% of hospitalized dengue patients, generally reflecting cytokine-mediated capillary leakage rather than primary cardiac disease (15). While most effusions are mild and self-limited, rare cases may deteriorate into tamponade (16), which can be difficult to distinguish from DSS given similar presentations of hypotension and respiratory distress. In both of our cases, pericardial effusion was part of widespread third-spacing rather than isolated cardiac inflammation. These findings underscore the importance of routine point-of-care ultrasonography in elderly DF patients with hemodynamic instability, as early identification of pericardial fluid can guide timely interventions and prevent misattribution to septic or dengue shock alone.

Patient 2 also highlights a relevant diagnostic pitfall: recent COVID-19 vaccination. Post-vaccination symptoms—fever, myalgia, malaise—overlap with early dengue manifestations, potentially delaying recognition of plasma leakage. Additionally, although rare, AP has been reported following vaccination (17), which may further obscure the etiology of abdominal symptoms. In endemic regions or during outbreaks, clinicians should maintain a high index of suspicion for DF even when symptoms appear temporally associated with vaccination.

Collectively, these cases emphasize that elderly patients with comorbidities represent a uniquely vulnerable population, likely to develop atypical and severe manifestations of severe dengue, characterized here as EDS involving AP and pericardial effusion. The overlap of systemic deterioration, bacterial coinfection, and metabolic derangements further complicates early diagnosis and timely management.

## Conclusion

These two cases highlight an uncommon but clinically significant presentation of DF—AP accompanied by pericardial effusion—in elderly patients with metabolic and cardiovascular comorbidities. Their atypical symptoms, delayed recognition, and rapid clinical deterioration demonstrate the need for heightened vigilance when evaluating older adults with nonspecific systemic complaints in dengue-endemic regions.

The novelty of this report lies in illustrating how dengue-associated AP and cardiac serositis can mimic other acute illnesses, coexist with bacterial coinfection, and present without typical abdominal pain, particularly in older adults. Early use of point-of-care ultrasound, careful interpretation of pancreatic enzyme elevation, and close hemodynamic monitoring are essential to differentiate dengue-related organ involvement from other comorbid conditions.

Future work should focus on identifying risk factors for dengue-associated AP in the elderly, clarifying the mechanisms underlying pancreatic and cardiac involvement, and developing diagnostic algorithms that integrate clinical, laboratory, and imaging findings to improve early recognition of these rare but serious complications.

## Data Availability

The original contributions presented in the study are included in the article/supplementary material. Further inquiries can be directed to the corresponding author.
